# Effect of short time exposure to magnetic on biology and DNA mutagenicity of the black cutworm *Agrotisipsilon *(Lepidoptera: Noctuidae)

**DOI:** 10.1038/s41598-025-95126-3

**Published:** 2025-04-13

**Authors:** Walaa H. Ahmed, Hanaa M. Ibrahim, Ghada E. Abd-Allah, Lina A. Abou El-Khashab

**Affiliations:** 1 Zoology and Entomology Department, Faculty of Science, Al-Azhar University (Girls Branch), Cairo, Egypt; 2 Agriculture Research Center, Plant Protection Research Institute, Giza, Dokki Egypt

**Keywords:** Magnetic fields, Biology, DNA mutagenicity, *Agrotisipsilon*, Molecular biology, Plant sciences, Zoology

## Abstract

*Agrotis ipsilon* is a significant pest of various crops, and the widespread use of chemical pesticides has led to numerous environmental issues, including pollution and the destruction of natural predators. Therefore, developing alternative pest control methods is essential. This study evaluates impact of short-term magnetic field exposure on the biological (life cycle duration) and genetic changes in *A. ipsilon* larvae. Larvae were exposed to a 180-milliTesla magnetic field for 20, 40, and 60 min using a Magnetizing Battery apparatus. The results showed that prolonged exposure significantly extended the larvae’s life cycle, with a 23.9% increase observed in the 60-min group compared to the control. Genetic analysis using Inter-Simple Sequence Repeat (ISSR) markers revealed mutagenic changes, with a polymorphism rate of 74.13%, indicating genetic alterations such as insertions and deletions. These findings suggest that magnetic fields may influence insect development and genetics, offering a potential alternative to chemical pesticides. Further research is needed to explore its broader applications in sustainable pest management to reduce environmental impacts.

## Introduction

The black cutworm *Agrotis ipsilon*(Hufnagel, 1766) (Lepidoptera: Noctuidae), is devastating and globally pervasive pest is renowned for its omnivorous feeding behavior and unparalleled capacity to inflict widespread agricultural destruction^1^.The larval stages of the black cutworm are particularly harmful, they cause substantial damage to many agricultural and horticultural crops particularly, at the seedling stage. This insect produces vitiating symptoms with a young stage (larvae) by feeding on the epidermis of leaves and eating away parts of the stem, tubers, etc.^2^. During development, these larvae can consume more than 400 cm^2^of foliage, highlighting their significant impact on agricultural productivity^3^.

Traditionally, chemical insecticides have been employed to mitigate crop losses caused by *A. ipsilon*. However, this approach had led to effect onnon-target organisms, such as beneficial insects and aquatic life leading to ecosystem imbalances and potential food chain contamination^4,5^. These negative consequences underscore the need for more sustainable and environmentally friendly pest management strategies. Additionally, insects have begun developing high levels of chemical pesticide resistance^6^.

There are more environmentally friendly pest control methods that address the problem of pesticide resistance, including biological control (application of entomopathogens, predators, and parasitoids)^7,8^and physical pest control^9^. Physical control methods, including magnetic fields, may provide new alternatives to chemical pesticides and promote eco-friendly insect pest management^10^.

Many biological processes in various organisms are affected by magnetic fields^10^including animals^11^, and insects^12,13^.These impacts involve behavior modifications^11^, development^14^, neural conduction^15^, and genetics^16,17^.

Genetic variation is an indicator of the short- and long-term survival of insect species after exposure to magnetic field. Accordingly, the application of MF is considered a promising method of insect control^12^reporting a direct effect of magnetic fields on the chitin of the insect cuticle. Exposure to MF has been shown to affect various insect species; cockroaches exposed to exhibit stress responses, increased motor activity, and delayed reactions to noxious heat stimuli. In locusts, MF exposure results in diminished kick force, reduced movement, and alterations in motor neuron activity. Crickets subjected to EMFs display changes in male calling song patterns and fluctuations in brain amine levels. MF exposure influences insect behavior and physiology in diverse ways, potentially affecting key processes like metamorphosis and metabolism^18,19^.

Accordingly, the application of MF is considered a promising method of insect control^12,13^. These effects included changes on molecular analysis and enzymatic activity in insects^20^. Limited researches had been conducted on the effects of magnetic fields on insect genetic changes.

These alterations can be detected as changes in the ISSR profile, which manifest as variations in band intensity or the presence or absence of specific bands^21^.

Several molecular marker techniques have been assessed in insect populations^22^.

In the context of this study, ISSR markers will be applied to examine the genetic effects of magnetic field exposure on *A. ipsilon*. Exposure to magnetic fields will induce biological and genetic changes in A. ipsilon larvae, leading to alterations in their development and DNA structure, including mutations such as insertions or deletions and rearrangements. These alterations can be detected as changes in the ISSR profile, which manifest as variations in band intensity or the presence or absence of specific bands. By utilizing ISSR markers, this study aims to identify and quantify these genetic changes, providing insights into how magnetic fields influence genetic variability in *A. ipsilon* and their implications for the insect’s adaptability and survival.

Limited research has been conducted on the effects of magnetic fields on insectsso this work provides a deeper understanding of magnetic field-induced effects on *A. ipsilon* and contributes to the development of sustainable pest control strategies that align with modern environmental and agricultural priorities.

The current study aims to investigate the biological and genetic effects of magnetic fields as a transformative tool in integrated pest managementon *A. ipsilon* larvae and monitor alterations in treated *A. ipsilon* larvae following exposure to magnetic fields for durations of 20, 40, and 60 min at biological and molecular level which helps to reduce the use of pesticides especially in Egypt.

## Materials and methods

### Insect rearing

*A. ipsilon*specimens were reared in a controlled environment with the following conditions: Temperature: 26 ± 1°C,relative humidity: 65 ± 5%, and photoperiod: Reversed 16:8 light: dark cycle^3^. All above conditions were carefully controlled and monitored to ensure no fluctuations during the experiment.

The rearing process was conducted as follows:

- 1. The freshly hatched larvae were put in clean 1-L glass jars.
- 2. Castor oil leaves were provided daily as a food source until the larvae reached the third instar.
- 3. To prevent cannibalism, third instar larvae were transferred to larger 2 L glass jars.
- 4. A dense layer of fine sawdust lined the bottom of each jar.
- 5. Standard rearing techniques were employed throughout larval development until pupation.
- 6. Upon emergence, adult moths were given a 10% sugar solution as a dietary supplement.

This rearing protocol was adapted from^23^.

### Magnetic field

The magnetic flux was measured using a magnetizing battery apparatus, which generated a magnetic field strength of 180 milliTesla (mT), as described by^24^This apparatus, provided by Prof. Dr. Abdel Khalek Hussein from the Plant Protection Research Institute. The apparatus which generates magnetic field (Patent 1663/ 2018, PPRI, ARC) consists of two components (rows). Each row consists of eight magnetic pieces, with 30 millitesla (mT) power each that are arranged in an attractive position. The two rows are placed parallel, with 2 cm in between, and in a repulsion position, generating a magnetic field (MF) of 180 mT.

This method ensured that any fluctuations in field strength remained within an acceptable margin of error, allowing for reliable and repeatable results.

### Bioassays and statistical analysis

Second instar larvaeof the black cutworms, *A. ipsilon*, were subjected to magnetic field for varying durations: 20, 40, and 60 min. Following exposure, the biological aspects.

(life cycle duration, larval longevity, and total life span) were monitored and recorded after exposure. These parameters were calculated for each exposure time.

To ensure the reliability and reproducibility of the results, all experiments were conducted with three biological replicates for each treatment group. Each replicate involved independent rearing and exposure of *A. ipsilon* larvae to the magnetic field, with subsequent analysis of biological and genetic parameters. Statistical analysis was performed using **CoStat software**^25^to assess the significance of differences among experimental groups using **ANOVA** (Analysis of Variance) followed by the LSD post-hoc test to evaluate pairwise comparisons with significance set at **p < 0.05**.

### Genomic DNA extraction

DNA was extracted from*A.ipsilon*4^th^ instar larvaeafter exposure to magnetic fields for 20, 40, and 60 minutesin the second instar larval stage,as well as from control larvae, utilizing the QIAampDNA Mini Kit (QIAGEN) based onthemanufacturer’s instructions. A UV spectrophotometer was used to measure the quality and concentration of the DNA solution by a ratio of A260/A280. Eleven ISSR primers were tested with treated and control larvae, as shown in Table 1.

**Table 1:** ISSR primer sequences.

No		Name	Seq
**1.**	**P1**	**UBC-824**	**TCTCTCTCTCTCTCTCG**
**2.**	**P2**	**17898A**	**(CA)6AC**
**3.**	**P3**	**17898B**	**(CA)6GT**
**4.**	**P4**	**HB 8**	**(GA)6GG**
**5.**	**P5**	**TA-2**	**(CT)10G**
**6.**	**P6**	**UBC-823**	**TCTCTCTCTCTCTCTCC**
**7.**	**P7**	**HB 10**	**(GA)6CC**
**8.**	**P8**	**TA-3**	**(AGG)6**
**9.**	**P9**	**UBC-844**	**CTCTCTCTCTCTCTCTRC**
**10.**	**P10**	**844A**	**(CT)8AC**
**11.**	**P11**	**17899A**	**(CA)6AG**

### ISSR polymorphism analysis

Polymerase chain reactions for ISSR analysis were performed in 25 μL volumes, according to^26,27^.Twenty ng of genomic DNA, 12.5 μL of Ferment as master mix, and 10 pmol of primer (Operon Technologies, Alameda, CA) were included in each reaction tube. A thermal cycler (TC96K, Acculab, USA) was used to perform the amplifications. It was first set to run for five minutes at 94°C for denaturation, then for 35 cycles at 94°C for one minute, 50°C for one minute, 72°C for ninety seconds, and 72°C for a final extension that lasted for seven minutes. A 2% agarose gel was used to visualize the amplified DNA, and a Gel Doc XR system (Bio-Rad, USA) was used to take pictures^28^.

### Construction of dendrogram based on ISSR-PCR DNA banding patterns

For ISSR data analysis, the bands were scored visually as absent (0) and present (1).

The polymorphism percentage was analyzed by dividing the number of amplified polymorphic bands by the total number of amplified bands separately for each primer^29^. A similarity matrix was built to estimate the genetic distances between all possible treatment pairs. The Jaccard coefficient was used for the pairwise comparisons^30^. Using NTSYS-pc and the unweighted pair group technique with arithmetic mean (UPGMA), clustering was done and a dendrogram was created^31^.

## Results

### Effect of different magnetic exposure times on the biology of larvae

Table 2 demonstrated that the incubation period, total larval duration, pupal duration, and overall life cycle increased with longer magnetic exposure times.

**Table 2:** Effect of different times of magnetization on the biology of *A. ipsilon.*

Time of magnetization	Incubation period	Average developmental periods of larval instars (in days)							Pre-pupa stage	Pupa stage	Life cycle
		1^st^ larval instar	2^nd^ larval instar	3^rd^ larval instar	4^th^ larval instar	5^th^ larval instar	6^th^ larval instar	Total larval stages			
20 min	**4 ± 0.05**	**2.2 ± 0.07**	**2.3 ± 0.07**	**2.3 ± 0.07**	**4 ± 0.09**	**5.2 ± 0.1**	**6.1 ± 0.2**	**22.1 ± 0.9**	**1.8 ± 0.1**	**20.2 ± 0.3**	**48.1 ± 1.1**
40 min	**4.2 ± 0.05**	**2.7 ± 0.09**	**2.9 ± 0.09**	**3 ± 0.08**	**4.3 ± 0.03**	**5.5 ± 0.09**	**6.4 ± 0.09**	**24.8 ± 0.6**	**1.9 ± 0.1**	**21.3 ± 0.3**	**52.2 ± 1.2**
60 min	**4.5 ± 0.05**	**3 ± 0.08**	**3 ± 0.08**	**3.2 ± 0.07**	**4.7 ± 0.07**	**5.9 ± 0.06**	**6.7 ± 0.06**	**26.5 ± 0.6**	**2 ± 0.1**	**24 ± 0.4**	**57 ± 1.3**
Control	**4.2 ± 0.05**	**2.5 ± 0.07**	**3 ± 0.07**	**3.5 ± 0.06**	**5 ± 0.06**	**4.8 ± 0.07**	**4 ± 0.07**	**22.8 ± 0.7**	**1.5 ± 0.1**	**17.5 ± 0.3**	**46 ± 1.1**
LSD at 0.05	**0.25**	**–––**	**–––**	**–––**	**–––**	**–––**	**–––**	**0.22**	**–––**	**0.81**	**0.19**

At 40 min, the incubation period of the eggs was the same as the control (4.2 days), but this period decreased after 20 min (4 days) and increased after 60 min (4.5 days) compared with the control. However, the total larval duration decreased after 20 min (22.1 days) but increased after 40 and 60 min (24.8 and 26.5 days, respectively) compared with the control (22.8 days). Furthermore, the total life cycle increased after 20, 40, and 60 min (48.1, 52.2, and 57 days, respectively) compared with the control (46 days).

Moreover, the magnetization time significantly affected the generation and longevity of both females and males, as represented in Table 3.

**Table 3:** Effect of time of magnetization on generation, longevity, and life span of *A.ipsilon.*

Time of magnetization	Duration of different adult stages (in days)						
	Female					Male	
	Pre-oviposition	Generation	Post-oviposition	Longevity	Life span	Longevity	Life span
20min	**11 ± 0.5**	**59.1 ± 1.2**	**4 ± 0.4**	**26 ± 1.1**	**74.1 ± 1.9**	**11 ± 0.6**	**59.1 ± 1.3**
40 min	**10 ± 0.6**	**62.2 ± 1.3**	**3 ± 0.3**	**20 ± 0.9**	**72.2 ± 1.8**	**9 ± 0.5**	**61.2 ± 1.4**
60 min	**––-**	**––-**	**––-**	**––-**	**––-**	**6 ± 0.4**	**63 ± 1.4**
Control	**7 ± 0.3**	**53 ± 1.1**	**2.5 ± 0.2**	**15.5 ± 0.8**	**61.5 ± 0.9**	**7 ± 0.3**	**53 ± 0.9**
L. SD at 0.05	**–––**	**0.13**	**––-**	**0.17**	**0.13**	**1.12**	**0.17**

Notably, after 60 min of magnetization, many treated larvae did not survive, and the remaining pupae developed into males. Male longevity after 60 min wassix days compared toseven days for the control, while the male life span was 63 days compared to 53 days for the control.

Additionally, the female generation increased after 20 and 40 min of magnetization, recording 59.1 and 62.2 days, respectively, compared to the control (53 days). Female longevity and life span increased compared to the control, recording 26, 74.1, and 20, 72.2 daysafter 20 and 40 min, respectively, compared to the control (15.5 and 61.5 days). Furthermore, male longevity and life span after 20 and 40 min increased significantly compared to the control, recording 11, 59.1, and 9, 61.2 daysafter 20 and 40 min, respectively, compared to the control (7 and 53 days).Furthermore, the magnetization time significantly affected the oviposition period, and the total average number of eggs decreased with increased magnetization time compared to the control. This effect is represented in Table 4**.**According to Table 4, the total average number of eggs decreased after 20 min of magnetization to 116.4 eggs, while exposure to 40 min led to a significant decline, with the total average number of eggs recorded at 45.3 eggs compared to 340 eggs for the control.

**Table 4:** Effect of time of magnetization on oviposition period and number of deposited eggs.

Time of magnetization	oviposition period (in days)	Number of deposited eggs	
		Total average	Daily mean
20 min	**11 ± 0.4**	**116.4 ± 8.6**	**10.6**
40min	**7 ± 0.3**	**45.3 ± 3.9**	**6.8**
60min	**––**	**––-**	**––-**
Control	**6 ± 0.3**	**340 ± 9.2**	**56.7**
L. SD at 0.05	**0.13**	**––-**	

### ISSR analysis

**Fig.1:**
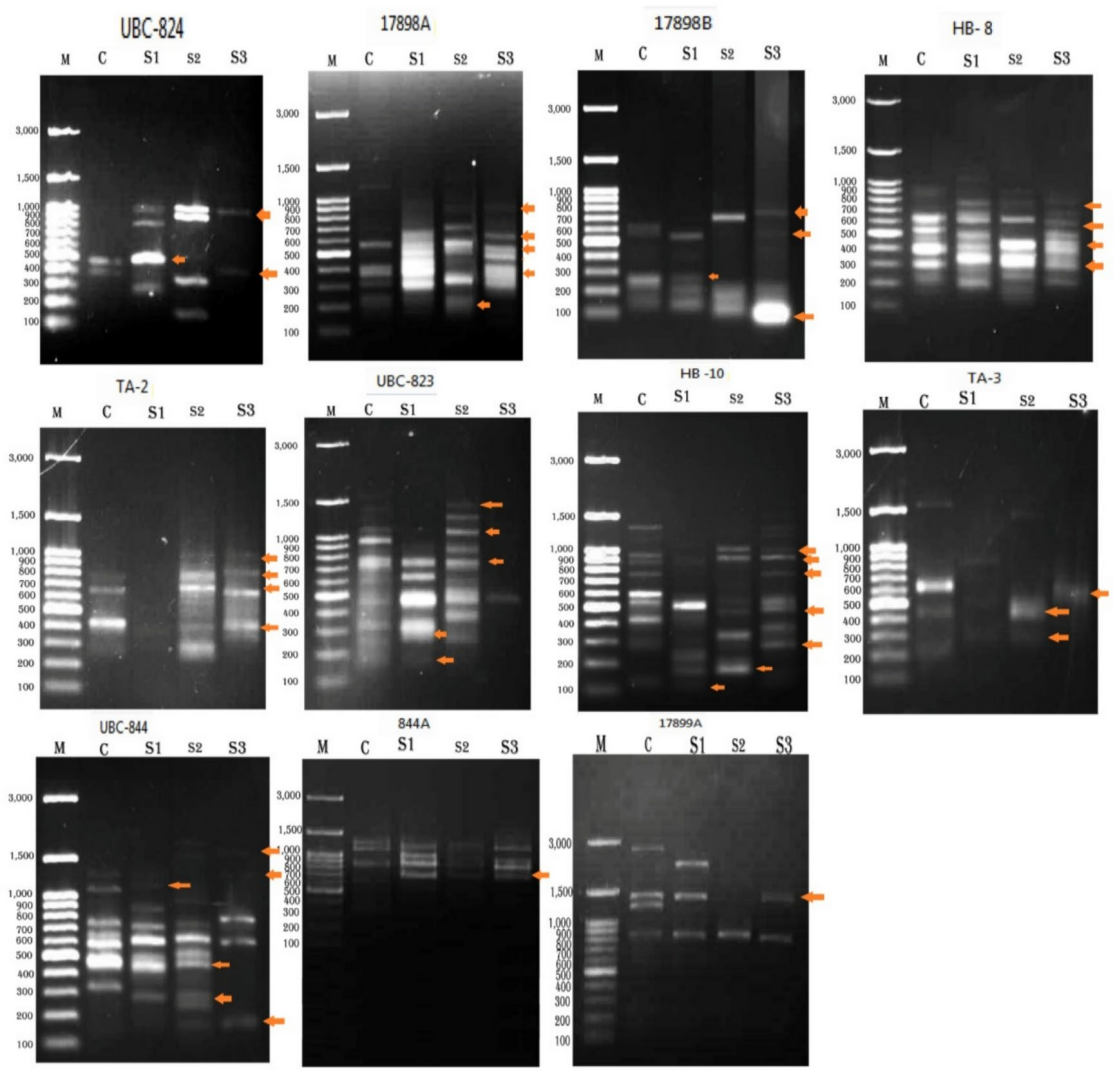
ISSRgel profiles of control and treated *A.ipsilon* larvae produced by using 11 primers. M: DNAmarker; C: Control; S1: Treated *A.ipsilon* larvae after 20 min; S2: Treated *A.ipsilon* larvae after 40 min; S3: Treated *A.ipsilon* larvae after 60 min, red arrows indicate polymorphic bands.

Total DNA extracted from control and treated *A.ipsilon* larvae of fourth instar after exposure to magnetic fields for 20, 40, and 60 min, analyzed on 2% agarosegel,represented high molecular weight DNA. A260/A280 analysis revealed a 20-ng concentration and 1.8 purity of DNA. Figure 1 shows the ISSR findings utilizing eleven primers for both control and treated larvae. eleven ISSR primers namely UBC-824, 17898A, 17898B, HB- 8, TA-2, UBC-823, HB −10, TA-3,UBC-844, 844A , and17899A as in Figs. 1&Table 5. In UBC-824 primer, the total bands were three with molecular weights (MW) of 1000, 500 and 400bp. All treated samples were different compared to control, also there were bands that disappeared from control. In 17898A primer, the total generated bands were seven with MW ranged from 900 to 220 bp. All treated larvae were different compared to control. In 17898B primer, the total generated bands were five which had MW ranged from 700 to 100 bp. All treated samples were different from control. In TA-2 primer, the total bands were five, their MW ranged from of 1000 to 400 bp. All treatments were different from control where some bands appeared and others disappeared in the different treatments, there were no bands appeared in S1 (after 20 min of exposure to magnetic). In UBC-823 primer, the total generated bands were six with MW ranged from 1400 to 200 bp, there was only one band appearing after 60 min of treatment. In HB −10 primer, the total generated bands were eight with MW ringing from 1000 to 140 bp. All treated samples were different from control. In TA-3 primer the total generated bands were three that had MW of 600,420 and 300 bp , all treated samples were different from control. UBC-844 primer showed eight bands ranging from 1600 to 150 bp, there were great differences between treated samples and control, In 844A primer there were three bands with MW of 1700, 800 and 700 bp, there were differences between treated samples and control. Also, 17899A primer showed variation between control and treated samples.Table 6 showed the monomorphic and polymorphic of larvae exposed to magnetic fields for different time 20, 40 and 60 h, compared with control. The data showed that total generated bands were 3, 7, 5, 8, 5, 6, 8, 3, 8, 3, and 2 bands in which monomorphic bands between treatments were 0, 2, 1, 4, 1, 1, 1,0, 2, 2, and 1 bands so the polymorphic band were 3, 5, 4, 4, 4, 5, 7, 3, 6, 1 and 1 bands, respectively. This means that polymorphism % were 100, 71.42, 80, 50, 80, 83.33, 87.5, 100, 75, 33.33 and 50 for the UBC-824, 17898A, 17898B, HB- 8, TA-2, UBC-823, HB −10, TA-3, UBC-844, 844A , and17899A primers, respectively, yielding 74.13% polymorphism among treated and control larvae. The highest level of polymorphism (100%) was observed with UBC-824 and TA-3 primers.

**Table 5:** ISSR profile of *A.ipsilon* larvae after exposure to magnetic fields in different duration time.

Primers	Band No	Treatments			
		C	S1	S2	S3
UBC-824	**1**	**0**	**1**	**1**	**1**
	**2**	**0**	**0**	**1**	**0**
	**3**	**0**	**1**	**0**	**0**
	**4**	**1**	**1**	**0**	**0**
	**5**	**1**	**0**	**0**	**1**
	**6**	**0**	**0**	**1**	**0**
	**7**	**0**	**1**	**0**	**0**
	**8**	**0**	**0**	**1**	**0**
	**Total**	**2**	**4**	**4**	**2**
17898A	**1**	**1**	**0**	**0**	**0**
	**2**	**0**	**0**	**1**	**1**
	**3**	**0**	**0**	**1**	**0**
	**4**	**0**	**1**	**1**	**1**
	**5**	**1**	**1**	**1**	**1**
	**6**	**0**	**1**	**1**	**1**
	**7**	**1**	**1**	**0**	**1**
	**8**	**1**	**1**	**1**	**1**
	**9**	**1**	**1**	**1**	**0**
	**Total**	**5**	**6**	**7**	**6**
17898B	**1**	**0**	**0**	**1**	**1**
	**2**	**1**	**0**	**0**	**0**
	**3**	**0**	**1**	**0**	**1**
	**4**	**0**	**1**	**0**	**0**
	**5**	**1**	**1**	**0**	**0**
	**6**	**1**	**1**	**1**	**1**
	**7**	**0**	**1**	**1**	**1**
	**Total**	**3**	**5**	**3**	**4**
HB 8	**1**	**0**	**1**	**0**	**0**
	**2**	**1**	**0**	**1**	**0**
	**3**	**0**	**1**	**1**	**1**
	**4**	**1**	**1**	**1**	**1**
	**5**	**1**	**0**	**0**	**1**
	**6**	**0**	**1**	**0**	**1**
	**7**	**1**	**1**	**1**	**1**
	**8**	**1**	**1**	**1**	**1**
	**9**	**1**	**1**	**1**	**1**
	**10**	**0**	**0**	**1**	**0**
	**Total**	**6**	**7**	**7**	**7**
TA-2	**1**	**0**	**0**	**1**	**1**
	**2**	**1**	**0**	**1**	**1**
	**3**	**1**	**0**	**1**	**1**
	**4**	**1**	**0**	**1**	**1**
	**5**	**0**	**0**	**1**	**0**
	**Total**	**3**	**0**	**5**	**4**
UBC-823	**1**	**1**	**0**	**0**	**0**
	**2**	**1**	**0**	**1**	**0**
	**3**	**0**	**0**	**1**	**0**
	**4**	**1**	**0**	**1**	**0**
	**5**	**1**	**0**	**0**	**0**
	**6**	**0**	**0**	**1**	**0**
	**7**	**1**	**1**	**1**	**0**
	**8**	**1**	**1**	**1**	**1**
	**9**	**0**	**0**	**1**	**0**
	**10**	**1**	**1**	**0**	**0**
	**11**	**0**	**0**	**1**	**0**
	**12**	**1**	**1**	**0**	**0**
	**Total**	**8**	**4**	**8**	**1**
HB 10	**1**	**1**	**0**	**0**	**0**
	**2**	**0**	**0**	**0**	**1**
	**3**	**0**	**0**	**0**	**1**
	**4**	**0**	**0**	**1**	**1**
	**5**	**1**	**0**	**1**	**1**
	**6**	**1**	**0**	**0**	**0**
	**7**	**1**	**0**	**0**	**1**
	**8**	**1**	**0**	**0**	**0**
	**9**	**1**	**1**	**1**	**1**
	**10**	**0**	**0**	**1**	**1**
	**11**	**1**	**0**	**0**	**0**
	**12**	**0**	**0**	**0**	**1**
	**13**	**0**	**0**	**1**	**0**
	**14**	**1**	**0**	**0**	**1**
	**15**	**0**	**1**	**0**	**0**
	**16**	**0**	**1**	**1**	**0**
	**17**	**1**	**1**	**0**	**0**
	**Total**	**8**	**4**	**6**	**9**
TA-3	**1**	**1**	**0**	**0**	**0**
	**2**	**0**	**0**	**1**	**0**
	**3**	**0**	**1**	**0**	**0**
	**4**	**1**	**0**	**0**	**1**
	**5**	**1**	**1**	**1**	**0**
	**6**	**0**	**1**	**1**	**0**
	**7**	**1**	**0**	**0**	**0**
	**Total**	**4**	**3**	**3**	**1**
UBC-844	**1**	**0**	**0**	**1**	**0**
	**2**	**0**	**0**	**1**	**1**
	**3**	**1**	**1**	**0**	**1**
	**4**	**1**	**1**	**0**	**0**
	**5**	**0**	**0**	**1**	**0**
	**6**	**0**	**1**	**0**	**0**
	**7**	**0**	**0**	**0**	**1**
	**total**	**2**	**3**	**3**	**3**
844A	**1**	**1**	**1**	**1**	**1**
	**2**	**0**	**0**	**1**	**0**
	**3**	**1**	**1**	**1**	**1**
	**4**	**0**	**1**	**1**	**1**
	**5**	**1**	**0**	**0**	**0**
	**Total**	**3**	**3**	**4**	**3**
17899A	**1**	**1**	**0**	**0**	**0**
	**2**	**0**	**1**	**0**	**0**
	**3**	**1**	**1**	**0**	**1**
	**4**	**1**	**0**	**0**	**0**
	**5**	**1**	**1**	**1**	**1**
	**Total**	**4**	**3**	**1**	**2**

**Table 6:** List of the ISSR primer names, their number of total bands, polymorphic bands, and percentage of polymorphism.

Primer Name	Total No. of Bands	Polymorphic Bands	Monomorphic Bands	Unique Band	% of polymorphism
UBC-824	3	3	-	5	100
17898A	7	5	2	2	71.42
17898B	5	4	1	2	80
HB 8	8	4	4	2	50
TA-2	5	4	1	-	80
UBC-823	6	5	1	6	83.33
HB 10	8	7	1	9	87.5
TA-3	3	3	-	4	100
UBC-844	8	6	2	7	75
844A	3	1	2	2	33.33
17899A	2	1	1	3	50
Total	58	43	15	42	74.13

### Data analysis

**Fig. 2:**
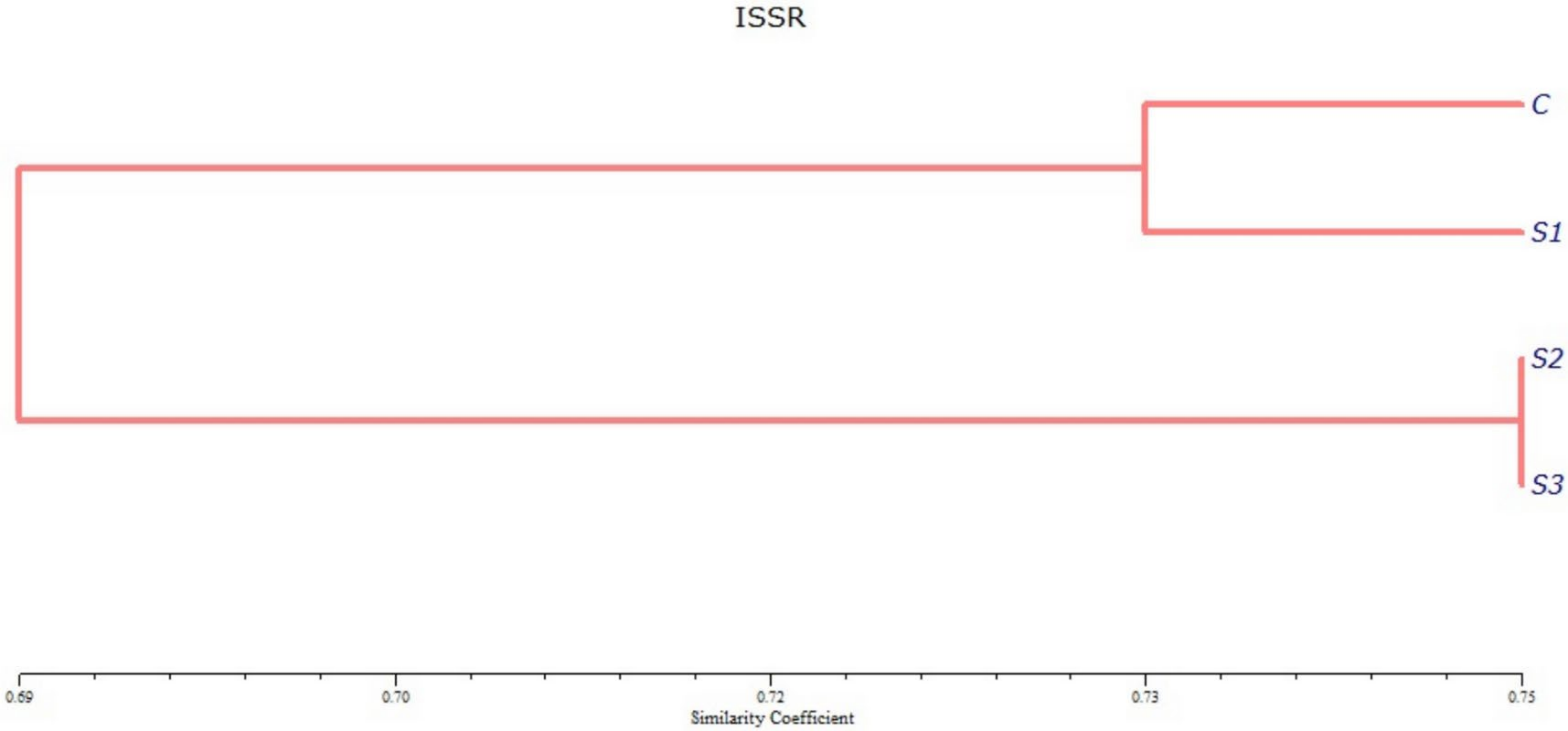
Dendrogram showing the result of cluster analysis between *A.ipsilon* larvae of 2^nd^ instar after exposure to magnetic fields for 20 (S1), 40 (S2), 60 (S3) minutes, and control ones (**C**) according to the ISSR analysis.

The similarity between *A.ipsilon* larvae after exposure to magnetic fields and control ones was showed in the dendrogram in Fig. 2. The tree was comprised of two clusters: one includes the control larvae and larvae treated with magnetic fields for 20 min (S1), while the other cluster includes larvae exposed to magnetic fields for 40 min (S2) and 60 min (S3).

The highest genetic similarity (GS) was observed between *A.ipsilon* larvae ofafter exposure to magnetic fields for 40 min (S2) and those exposed for 60 min (S3), followed by the high genetic similarity between the control (C) and larvae exposed to magnetic fields for 20 min (S1). The lowest GS was found between the control and larvae exposed to magnetic fields for 40 min (S2) and 60 min (S3) (Table 7**).**

**Table 7:** Genetic similarities between treated *A.ipsilon* and untreated larvae according to Jaccard’s similarity coefficient for ISSR analysis.

	C	S1	S2	S3
C	100%			
S1	73. 48%	100%		
S2	65. 91%	71. 21%	100%	
S3	66. 67%	71. 97%	75%	100%

## Discussion

This study assessed the impacts of magnetic fields on *A.ipsilon* larvae, focusing on biological and genetic changes; this is because larvae are the harmful phase in the life of *A.ipsilon*,the results demonstrated that the incubation period, total larval, pupal duration, and overall life cycle(23.9%) increased with longer magnetic exposure times 60-min group compared to the control. The results obtained agree with those of many investigators^32,33^. Also, the results in this studyconsistent with the finding of^33^who applied a magnetic field to *E. cautella*for varying durations (10, 20 and 30 min) and measured biological parameters (Egg incubation duration, larval stage duration, pupal duration, adult longevity and life cycle duration from egg to adult). The total life cycle duration increased following exposure of eggs to the magnetic field, this may be due to the effect of the magnetic field on proteins and enzyme during life cycle. Also, in the study of^13^the exposure *E. insulana* adults was performed with the effects of two magnetic levels, indicated that the time require to the eggs of spiny bollworm hatching was high significant affected by two treatment of high and low magnetic fields.

However^34^ reported that all biological parameters were negatively affected by application of a magnetic field for 4 h during the rearing of the larvae of the rice moth, *C. cephalonica* but the application of the magnetic field for 2 h. had beneficial effects.

This might be due to MF exposure can decreases the biological functions of insect proteins^13^and produces genetic variations^16,17^^.^Which may influence developments process^18,19^, in addition to deactivates enzymatic active sites,

Moreover, in this study the magnetization time significantly decreased the generation and longevity of both females and males^35^. used a moderate intensity SMF (0, 1.5, 3, 5, 7.5 and 10 mT) to detect its biological effects on *E. kuehniella*adults. The longevity of adults, fecundity and daily egg production have highly decreased. Similarly^36^found a high effect of the magnetic on behavior and longevity of *Tenebrio molitor*, In addition^37^, recorded high effect of magnetic on fecundity and longevity on aphid^32^. also observed that, longevity of *Tetranychusurticae*was affected by magnetic fields. This may occur due to that magnetic fields minimize populations of harmful insects and might delay egg development^31^.

Additionally, the results also, showed that the total average number of eggs significantly decreased after 20 and 40 min of magnetization compared to that of control.

These findings in agreement with^13^ who illustrated that the total eggs laid per female and hatchability in exposed adults were highly significant decreased in comparison with control when eggs of *E. insulana*were exposed to a magnetic field. In addition^38^, observed a reduction in the number of eggs laid by pink bollworm females after prolonged exposure to magnetic fields, a result supported by^39^ who recorded high significant effect of three levels of magnetic flux on reduction of eggs laid by *P. gossypiella* and *E. insulana*. These observations may result because of up-regulatory changes or exposure to the magnetic field which may cause the denaturation of double-stranded DNA, and organisms may have activated DNA ligase for the repair^40^, this may lead to the general suppression of the metabolic and developmental processes.

In this research using Inter-Simple Sequence Repeat (ISSR) markers revealed mutagenic changes, with a polymorphism rate of 74.13%, indicating genetic alterations such as insertions and deletions. The results indicated that there were great DNA alterations between control and treated larvae, hence some bands appeared and others disappeared in different treatments compared to control for all primers that has been used. These may be due to effect of magnetic fields on alterations in primer binding sites or DNA structures as a result of blocked DNA replication, chromosomal lesions (manifesting as inversions, deletions, or translocations), or unrepaired DNA damage because of direct exposure^41,42^. demonstrated the relatively strong biological effects of MFs on the modulation of ion fowl interference with DNA synthesis and RNA transcription thus, affecting the genetic changes.

The 0, 1 matrices compared the amplification profiles of each primer and assigning a score of either present (1) or absent (0), showed that the highest level of polymorphism (100%) was observed with primers UBC-824 and TA-3. From these results, it may be stated that ISSR primer UBC-824 and TA-3 could be used to distinguish between all different treated larvae and magnetic fields affected on the DNA of the larvae, this change in DNA may be the reasonof toxicity of larvae due to magnetization. The ISSR had been widely used in experimental procedures. Previous research found that ISSR markers are helpful for qualitatively assessing DNA variability via variations in amplification profiles. Consistent with our results^18^, reported that different insecticide concentration levels caused changes in the genomic DNA sequences of the western bean cutworm, resulting in alterations in the ISSR profile and band intensity. The disappearance of bands may be attributed to alterations at the oligonucleotide annealing site, such as point mutations. Similarly^43^ used 15 ISSR primers to detect genetic variability levels and determine the degree of polymorphism between *A. ipsilon* larvae treated with nanoparticle insecticides and control larvae. Following the application of two distinct bioinsecticides to *Spodoptera littoralis*^44^, reported comparable outcomes. DNA replication and repair efficiency as well as the degree of DNA damage are closely correlated with genetic similarity (GS %). Also^45^, estimated DNA concentration and DNA damage for treated *Rhyzoperthadominica*exposed to some plant volatile oils.As suggested by^46^, ISSR markers were used to determine genotype of *A. lepigone* collected from 15 geographic locations in North China. Data from seven primers resulted in a total of 183 bands.

Additionally, ISSR markers helped identify several species of necrophagous flies from 12 cities in China^47^. Genetic relationships between mutant silkworm strains of *Bombyx mori*were detected using ISSR markers^48^.ISSR primers can target different genome regions and have great potential for recovering intra- and inter-genomic relationships. The UPGMA-based dendrogram showed similarities and variations among the tested samples and control, these variations may be due to prolonged exposure to the magnetic field may lead to cause the denaturation of double-stranded DNA. Thus, this study provides evidence Magnetic field exposure significantly increased the life cycle duration of *A. ipsilon* larvae and induced genetic polymorphisms, with 74.13% polymorphism observed across all treatments, also the effect of the magnetic field on the DNA in the hemolymph of *Bombyx mori*was investigated^49^. In addition^50^, reports that the RNA indicates an index of the capacity of protein synthesis and DNA contents help in the estimation of cell number. The protein synthesis of each cell is reflected by the RNA/DNA ratio^51^.

## Conclusion

This study provides valuable insights into the biological and genetic effects of magnetic fields on *A. ipsilon* larvae, revealing their potential application in sustainable integrated pest managementto reduce reliance on chemical pesticides. The use of ISSR markers successfully identified genetic mutagenicity and polymorphism induced by magnetic exposure, with higher exposure durations (40 and 60 min) resulting in significant mutagenic effects. These findings emphasize the role of magnetic fields as an effective environmental stressor that can induce genetic changes in insects, providing a novel approach to pest control. Future studies should focus on exploring the long-term effects of magnetic field exposure to insect populations, particularly in terms of reproductive success, resistance development, and ecological consequences. Additionally, expanding research to investigate the mutagenic effects of magnetic fields in other pest species would offer valuable insights into the broader applicability of this approach. Future research should investigate the effects of magnetic fields on all life stages of *A. ipsilon*, understanding the underlying molecular mechanisms, including potential DNA repair processes and gene expression changes, will be crucial in optimizing magnetic field treatments for pest control.

## Supplementary Information

Supplementary Information.

## Data Availability

All data generated during this study are included in this published article.
